# Clinical implication of initial intravenous diuretic dose for acute decompensated heart failure

**DOI:** 10.1038/s41598-022-06032-x

**Published:** 2022-02-08

**Authors:** Kenji Yoshioka, Daichi Maeda, Takahiro Okumura, Keisuke Kida, Shogo Oishi, Eiichi Akiyama, Satoshi Suzuki, Masayoshi Yamamoto, Akira Mizukami, Shunsuke Kuroda, Nobuyuki Kagiyama, Tetsuo Yamaguchi, Tetsuo Sasano, Akihiko Matsumura, Takeshi Kitai, Yuya Matsue

**Affiliations:** 1 Department of Cardiology, Kameda Medical Center, Chiba, Japan; 2 Department of Cardiovascular Medicine, Tokyo Medical and Dental University, Tokyo, Japan; 3 Department of Cardiovascular Medicine, Juntendo University, Tokyo, Japan; 4 Department of Cardiology, Osaka Medical and Pharmaceutical University, Osaka, Japan; 5 Department of Cardiology, Nagoya University Graduate School of Medicine, Nagoya, Japan; 6 Department of Pharmacology, St. Marianna University School of Medicine, Kawasaki, Japan; 7 Department of Cardiology, Himeji Cardiovascular Center, Hyogo, Japan; 8 Division of Cardiology, Yokohama City University Medical Center, Yokohama, Japan; 9 Department of Cardiovascular Medicine, Fukushima Medical University, Fukushima, Japan; 10 Cardiovascular Division, Faculty of Medicine, University of Tsukuba, Tsukuba, Japan; 11 Heart and Vascular Institute, Cleveland Clinic, Cleveland, OH USA; 12 Department of Cardiology, The Sakakibara Heart Institute of Okayama, Okayama, Japan; 13 Department of Cardiovascular Biology and Medicine, Juntendo University Faculty of Medicine, Tokyo, Japan; 14 Department of Digital Health and Telemedicine R&D, Juntendo University, Tokyo, Japan; 15 Department of Cardiology, Cardiovascular Center, Toranomon Hospital, Tokyo, Japan; 16 Department of Cardiovascular Medicine, National Cerebral and Cardiovascular Center, Osaka, Japan; 17 Cardiovascular Respiratory Sleep Medicine, Juntendo University Graduate School of Medicine, Juntendo University School of Medicine, Tokyo, Japan

**Keywords:** Heart failure, Heart failure

## Abstract

Although intravenous diuretics is a cornerstone of acute heart failure treatment (AHF), its optimal initial dose is unclear. This is a *post-hoc* analysis of the REALITY-AHF, a prospective multicentre observational registry of AHF. The initial intravenous diuretic dose used in each patient was categorised into below, standard, or above the recommended dose groups according to guideline-recommended initial intravenous diuretic dose. The recommended dose was individualised based on the oral diuretic dose taken at admission. We compared the study endpoints, including 60-day mortality, diuretics response within six hours, and length of hospital stay (HS). Of 1093 patients, 429, 558, and 106 were assigned to the Below, Standard, and Above groups, respectively. The diuretics response and HS were significantly greater in the Below group than in the Standard group after adjusting for covariates. Kaplan–Meier analysis indicated a significantly higher incidence of 60-day mortality in the Above group than the Standard group. This difference was retained after adjusting for other prognostic factors. Treatment with a lower than guideline-recommended intravenous diuretic dose was associated with longer HS, whereas above the guideline-recommended dose was associated with a higher 60-day mortality rate. Our results reconfirm that the guideline-recommended initial intravenous diuretic dose is feasible for AHF.

## Introduction

Decongestion with intravenous (IV) diuretics is a mainstay of acute heart failure (AHF) treatment since congestion is one of the primary reasons for heart failure admission^1^. Although diuretics are an effective treatment for most patients with AHF, the ideal dose of IV loop diuretics has yet to be established. The Diuretic Optimization Strategies Evaluation (DOSE) trial provided important insights regarding clinical and prognostic implications of high vs. low dose loop diuretics, finding no prognostic differences. However, current guidelines recommend using the smallest amount of diuretics to provide adequate decongestion^2,3^. This reflects the fact that a greater amount of loop diuretics tends to induce a stronger diuresis and greater relief of symptom. However, giving a large amount of diuretics is not always good given the adverse effects reported in patients with heart failure^4,5^. Current guidelines recommend 20 to 40 mg of IV furosemide for patients with AHF not receiving oral diuretics, or an equivalent or higher dose than the oral diuretics for those already taking it. However, these recommendations have not been validated yet^2,3^. Therefore, we sought to examine the current recommendations on the initial IV furosemide dose administered to patients with AHF in terms of treatment efficiency and prognostic impact, using the REALITY-AHF (Registry Focused on Very Early Presentation and Treatment in Emergency Department of Acute Heart Failure) cohort^6^.

## Results

Of the 1,682 patients enrolled in the REALITY-AHF, 1109 remained after excluding those not treated with furosemide within six hours of admission or treated with continuous furosemide infusion. We further excluded 26 patients with missing data on the amount of the first IV furosemide bolus. Consequently, 1093 patients were analysed (Supplemental Figure S1). These patients were assigned to one of three groups according to the guideline-recommended dose: Below (*n* = 429), Standard (*n* = 558), and Above (*n* = 106). Baseline characteristics of the three groups are shown in Table 1. Significant between group differences were observed in blood pressure, presence of orthopnoea and pulmonary oedema, history of heart failure, being treated with loop diuretics, beta-blockers, and/or aldosterone blocker before admission, and levels of haemoglobin, creatinine, and blood urea nitrogen. Of note, the Below group, but not the Above group, showed patient characteristics associated with poor prognosis such as lower systolic blood pressure, less orthopnoea and pulmonary oedema, more patients with a history of heart failure, being treated with high-dose loop diuretics before admission, and poor renal function in comparison to the Standard group.

**Table 1:** Baseline characteristics of the study participants.

Variables	Below *N* = 429	Standard *N* = 558	Above *N* = 106	*P*-value
Age (years)	78 (12)	78 (11)	79 (12)	0.522
Male sex (%)	246 (57.3)	303 (54.3)	56 (52.8)	0.546
Systolic blood pressure (mmHg)	146 (35)	158 (34)	157 (38)	< 0.001
Diastolic blood pressure (mmHg)	83 (24)	88 (26)	87 (29)	0.001
Heart rate (bpm)	96 (26)	102 (30)	95 (26)	0.003
Symptom onset time				0.426
≤ 6 h	98 (22.8)	147 (26.3)	26 (24.5)	
6 h–2 days	87 (20.3)	123 (22.0)	27 (25.5)	
> 2 days	244 (56.9)	288 (51.6)	53 (50.0)	
ECG rhythm (%)				0.021
Sinus	219 (51.2)	316 (56.6)	70 (66.7)	
AF	173 (40.4)	189 (33.9)	31 (29.5)	
Others	36 (8.4)	53 (9.5)	4 (3.8)	
LVEF at ED (%)				0.294
< 35%	158 (39.8)	171 (33.5)	31 (32.0)	
35–50%	113 (28.5)	166 (32.5)	34 (35.1)	
> 50%	126 (31.7)	173 (33.9)	32 (33.0)	
Physical examination (%)				
JVD	249 (59.3)	353 (64.3)	74 (71.2)	0.054
Orthopnoea	260 (60.9)	384 (68.9)	76 (71.7)	0.013
Rale	298 (69.8)	398 (71.3)	81 (76.4)	0.401
Peripheral oedema	308 (71.8)	398 (71.5)	84 (79.2)	0.246
Pulmonary oedema	317 (73.9)	445 (79.7)	87 (82.1)	0.047
Comorbidities (%)				
History of Heart Failure	269 (62.7)	222 (39.8)	60 (56.6)	< 0.001
Hypertension	286 (66.7)	388 (69.5)	71 (67.0)	0.608
Diabetes mellitus	161 (37.5)	193 (34.6)	45 (42.5)	0.260
COPD	41 (9.6)	60 (10.8)	8 (7.5)	0.561
Coronary artery disease	144 (33.6)	162 (29.0)	33 (31.1)	0.312
Medication at admission (%)				
Loop diuretics	288 (67.1)	183 (32.8)	65 (61.3)	< 0.001
Loop diuretics dose among takers (mg)	40 [40–60]	20 [20–20]	10 [10–20]	< 0.001
ACE-I	79 (18.4)	81 (14.5)	22 (20.8)	0.130
ARB	143 (33.3)	169 (30.3)	32 (30.2)	0.567
Beta blocker	216 (50.9)	195 (35.0)	38 (35.8)	< 0.001
Aldosterone blocker	117 (27.3)	80 (14.3)	20 (18.9)	< 0.001
Laboratory data at admission				
White blood cell (/µL)	7200 [5500–9900]	8000 [6000–10,400]	8100 [5925–10,675]	0.008
Albumin (g/dL)	3.47 (0.57)	3.46 (0.52)	3.42 (0.49)	0.716
Haemoglobin (g/dL)	11.6 (2.26)	12.0 (2.34)	11.7 (2.08)	0.007
AST (IU/L)	33 [22–45]	31 [23–46]	30 [24–58]	0.806
ALT (IU/L)	21 [14–33]	22 [14–36]	21 [13–36]	0.557
Creatinine (mg/dL)	1.20 [0.87–1.64]	1.02 [0.78–1.44]	1.15 [0.81–1.92]	< 0.001
BUN (mg/dL)	26 [19–37]	23 [17–31]	25 [19–35]	< 0.001
Sodium (mEq/L)	139 [137–142]	140 [137–142]	140 [137–142]	0.422
Potassium (mEq/L)	4.21 (0.63)	4.28 (0.71)	4.50 (0.81)	0.001
Glucose (mg/dL)	163 (76)	169 (76)	184 (90)	0.049
CRP (mg/dL)	0.56 [0.20–2.01]	0.77 [0.21–2.26]	0.84 [0.32–2.57]	0.115
BNP (pg/mL)	757 [439–1510]	707 [437–1254]	827 [409–1572]	0.373
Total furosemide used within six hours (mg)	20 [10–30]	20 [20–37]	40 [23–50]	< 0.001
Urine output within 6 h (mL)	755 [465–1168]	900 [580–1440]	980 [480–1370]	< 0.001
Catecholamines within 6 h (%)	39 (11.9)	49 (9.5)	17 (16.8)	0.085

Continuous variables are expressed as mean (standard deviation) or median [interquartile range].

*ACE-I* angiotensin-converting enzyme inhibitor, *AF* atrial fibrillation, *ALT* alanine aminotransferase, *ARB* angiotensin II receptor antagonist, *AST* aspartate aminotransferase, *BNP* brain natriuretic peptide, *BUN* blood urea nitrogen, *COPD* chronic obstructive pulmonary disease, *CRP* C-reactive protein, *ECG* electrocardiogram, *ED* emergency department, *JVD* jugular vein distention, *LVEF* left ventricular ejection fraction.

**Figure 1:**
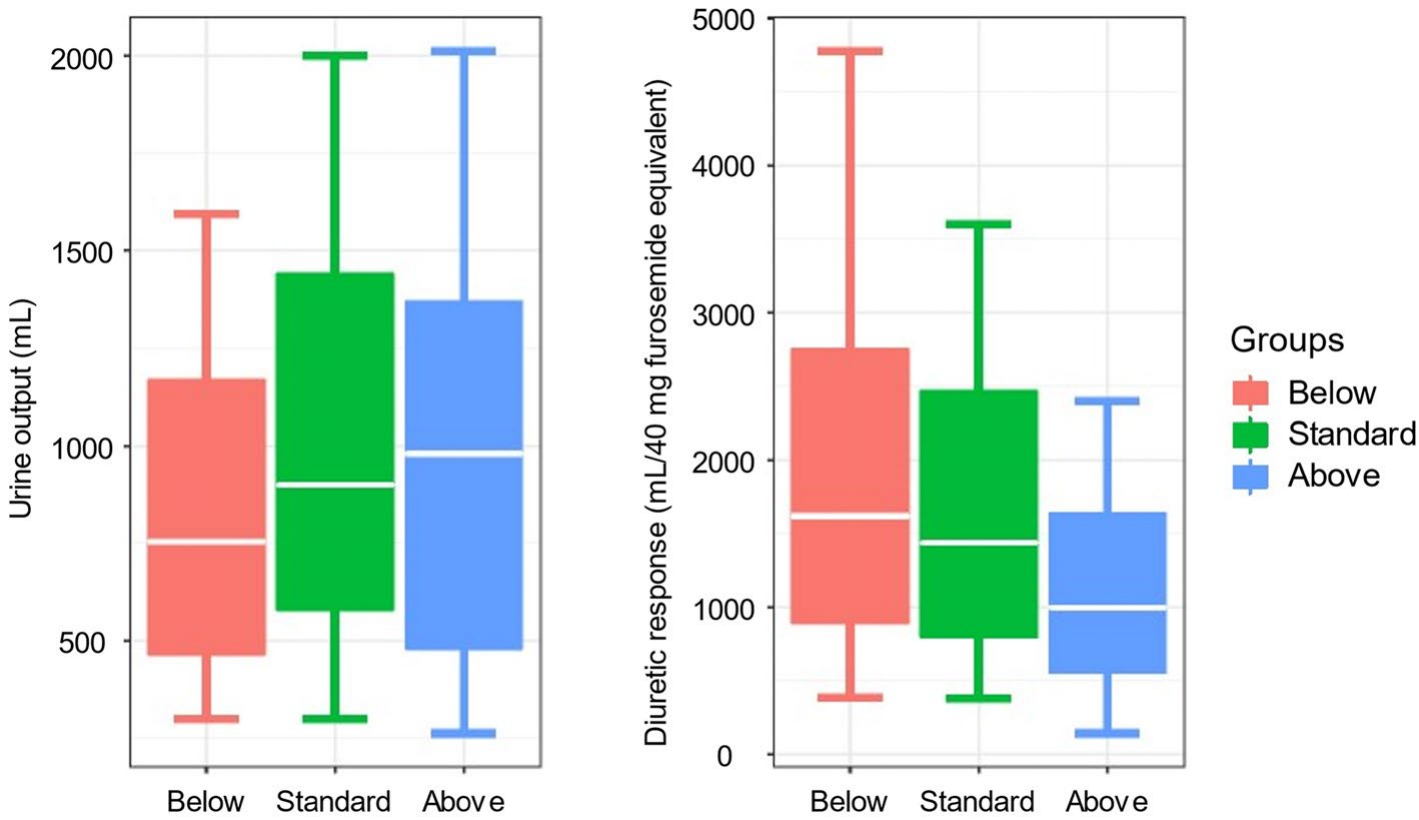
Urine output during the first six hours and diuretic response. Diuretic response was defined as the urine volume during the first six hours per 40 mg of IV furosemide. Although the urine output during the first six hours in the Above group was significantly larger than in the Standard group, its diuretic response was significantly lower (*P* < 0.001 for both).

The total amount of IV furosemide used within the first six hours of admission differed significantly between the Below (20 mg; interquartile range [IQR], 10–30 mg), Standard (20 mg; IQR, 20–37 mg), and Above (40; IQR, 23–50 mg) groups (*P* < 0.001). Urine output measured during the first six hours of admission and diuretic response (DR) are shown in Fig. 1. The urine output within during the six hours of admission was significantly higher and DR significantly lower in the Above group (*P* < 0.001 for both). Univariate linear regression analysis showed that the DR in the Above group was significantly lower and in the Below group significantly higher than in the Standard group (Table 2). After adjusting for covariates shown to be related to DR within six hours, the DR in the Below group remained significantly higher than the Standard group, whereas the Above and Standard groups were no longer statistically different.

**Table 2:** Association between dose groups and diuretic response.

Groups	Unadjusted model			Adjusted model*		
	Beta coefficient	95% CI	*P*-value	Beta coefficient	95% CI	*P*-value
Standard	Reference			Reference		
Below	358.6	127.4 to 589.6	0.002	371.1	109.5 to 632.8	0.006
Above	−612.5	−997.5 to −227.5	0.002	−296.6	–686.2 to 92.9	0.135

*Adjusted for age, whether were taking oral loop diuretics before admission, white blood cell count, and levels of serum albumin, creatinine, potassium, and brain natriuretic peptide.

*CI* confidence interval.

**Figure 2:**
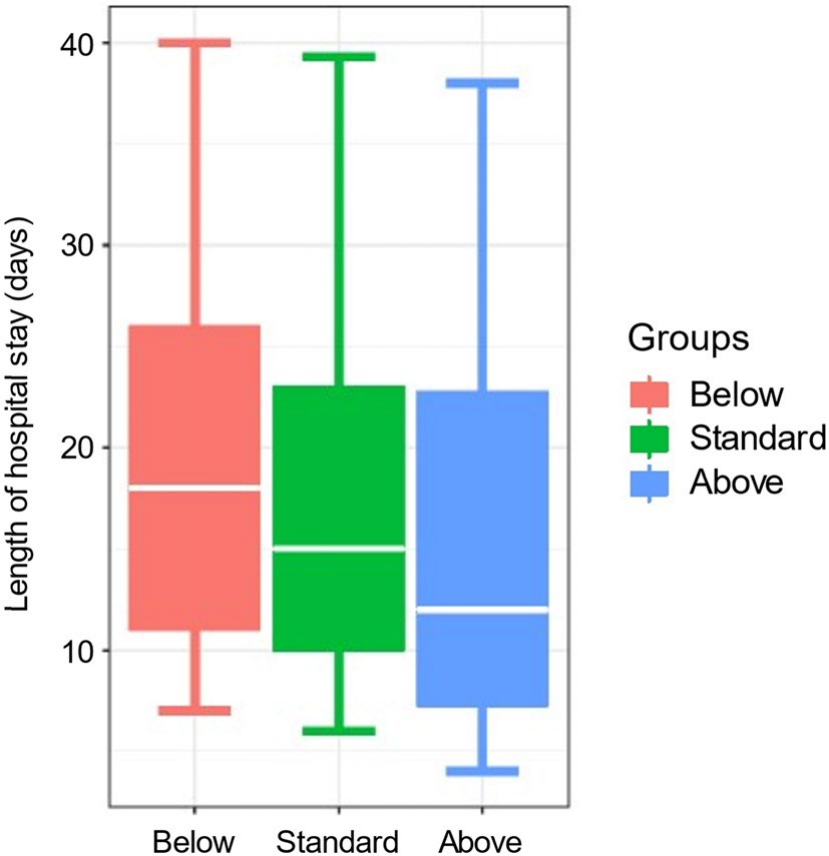
Length of hospital stay according to the first furosemide IV dose. The hospital stay in the Below group was significantly longer than in the other groups (*P* < 0.001).

The median length of hospital stay (HS) differed significantly between the Below (18 days; IQR, 11–26 days), Standard (15 days; IQR, 10–23 days), and Above (12 days; IQR, 7–22 days) groups (*P* < 0.001; Fig. 2). Multiple linear regression analysis showed that the Below group had a significantly longer HS than in the Standard group (Table 3), while HS in the Above group was marginally but insignificantly shorter than in the Standard group.

**Table 3:** Association between the dose groups and the length of hospital stay.

Groups	Unadjusted model			Adjusted model*		
	Beta coefficient	95% CI	*P*-value	Beta coefficient	95% CI	*P*-value
Standard	Reference			Reference		
Below	2.30	0.15 to 4.46	0.036	2.34	0.01 to 4.67	0.049
Above	−1.63	−5.26 to 1.20	0.376	−3.29	−7.02 to 0.44	0.084

Those who died during the index hospitalisation were excluded.

*BNP* brain natriuretic peptide, *CI*, confidence interval.

*Adjusted for age, sex, history of heart failure, and atrial fibrillation, the New York Heart Association class, systolic blood pressure, haemoglobin, serum creatinine, sodium, albumin, and log-transformed BNP.

**Figure 3:**
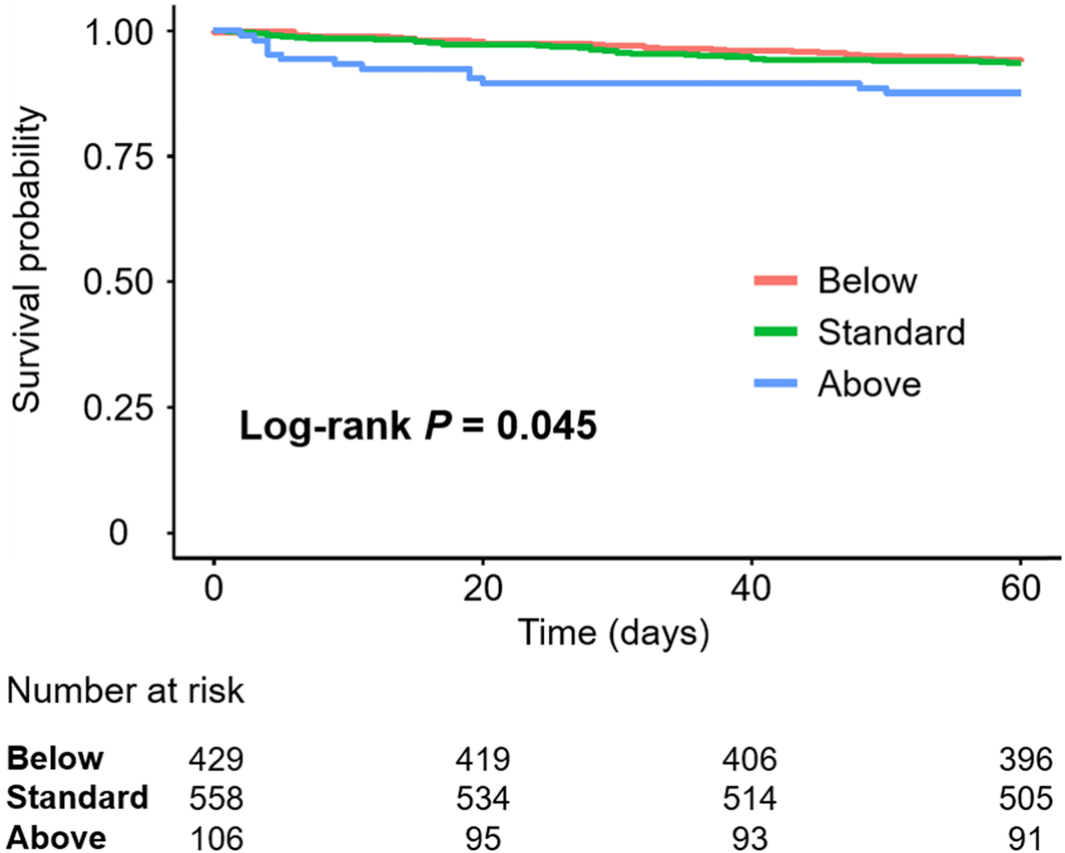
Kaplan–Meier curves for 60-day mortality according to the first furosemide IV dose. The Above group was significantly associated with a lower survival rate.

Seventy-four deaths were observed during the 60 days of admission. Kaplan–Meier curves showed that the Above group was significantly associated with a higher 60-day mortality rate (Fig. 3). The Cox regression analysis showed that the Above, but not the Below, group was associated with a significantly higher 60-day mortality rate than the Standard group in unadjusted and adjusted models (Table 4). As we have already demonstrated that the door-to-furosemide time was associated with the 30-day mortality ^6^, we added the door-to-furosemide time in the multivariable analysis. Furthermore, additional treatments such as the usage rate of vasodilators, catecholamines, and renin angiotensin aldosterone system inhibitors during the first 48 h were also included; however, the results remained unchanged (Table 4).

**Table 4:** Cox proportional hazard analysis for 60-day mortality according to initial furosemide dose groups.

	Unadjusted			Adjusted for model 1*			Adjusted for model 2**		
	HR	95% CI	*P* value	HR	95% CI	*P* value	HR	95% CI	*P* value
Dose groups									
Standard	1 (Reference)			1 (Reference)			1 (Reference)		
Below	0.95	0.57–1.58	0.842	1.01	0.48–2.13	0.980	1.18	0.53–2.62	0.692
Above	2.05	1.09–3.88	0.027	3.89	1.70–8.88	0.001	3.11	1.29–7.49	0.011
OPTIME-CHF score (per 1 point)				1.01	1.01–1.02	< 0.001	1.01	1.01–1.02	0.070
D2F time							1.00	1.00–1.00	0.977

*Adjusted for the OPTIME-CHF score and log-transformed brain natriuretic peptide.

**Adjusted for Model 1 plus door to furosemide time, dobutamine use within 48 h, dopamine use within 48 h, norepinephrine use within 48 h, phosphodiesterase III inhibitor use within 48 h, vasodilator use within 48 h, total furosemide used within 48 h, and angiotensin-converting enzyme inhibitor/angiotensin II receptor antagonist use within 48 h.

*CI* confidence interval, *D2F* door to furosemide, *HR* hazard ratio.

## Discussion

This study demonstrated that the Standard group, receiving the guideline-recommended initial diuretic IV dose, was associated with a shorter HS than in the Below group, and a higher 60-day survival rate than in the Above group, even after adjusting for various confounders. To the best of our knowledge, this is the first study to validate the guideline-recommended initial IV loop diuretic dose in an AHF cohort.

DR was recently suggested as a metric of diuretic efficiency, and poor DR was reported to be an independent predictor of worse outcomes in patients with AHF^7,8^. It is well known that dose–response curves of loop diuretics have a ceiling effect, suggesting that increasing the doses above a certain point will not increase the diuretic effect^9^. This effect was clearly shown in our study. Our results showed that higher-than-suggested furosemide doses were associated with a significantly poorer DR. The use of a higher diuretic dose is a trade-off between achieving urine output and the subsequent decongestion and risking the downsides of diuretic use. Doses above the ceiling level could be more harmful than beneficial. On the other hand, doses below the diuretic dose recommended by guidelines had significantly greater DR; however, smaller doses were not associated with a better 60-day prognosis than the standard dose and were associated with a longer HS. This finding might suggest that lowering the diuretic dose will not directly lead to a better prognosis, even with a greater DR. Rather, it would result in longer HS, possibly because of the lower urine output achieved. Indeed, the HS after AHF in Japan was reported to be longer than in Western countries. One of the reasons for this difference could be that the diuretic doses given to patients with AHF in Japan are lower than in Western countries^10,11^. As it happens, the guideline-recommended furosemide dose is the suitable one, neither too high nor too low.

The DOSE trial, a prospective, double-blind, randomised controlled trial focusing on the usage of IV furosemide in patients with AHF, demonstrated conflicting results. That study found no statistical differences between using low and high doses in terms of 60-day composite mortality and rates of re-hospitalisation and emergency visits for AHF^5^. Although the current recommendations on the initial IV diuretic dose were primarily derived from the DOSE study, we could not simply compare the DOSE and our studies because they differ in some crucial elements. First, the DOSE trial enrolled patients with a history of chronic heart failure that took oral loop diuretics equivalent to 80 to 240 mg furosemide, while the present study enrolled consecutive patients with AHF irrespective of whether the presented a de novo disease or were with chronic heart failure, and we did not limit the oral furosemide dose. Given that around half of the patients with AHF are with a newly diagnosed disease, our study seemed to better represent the real-world AHF population, making our results more clinically applicable. Second, the time from hospital arrival to IV furosemide use was quite different as well. We only enrolled patients who received the initial IV furosemide bolus within six hours of admission, whereas the patients in the DOSE trial were enrolled following a median time of 14.6 h from arrival, and the median duration of study-drug administration was 65.3 h. Besides, most patients in the DOSE trial received the initial IV diuretics after arrival and before enrolment. Third, the IV furosemide dose regimen was different. A bolus infusion of furosemide was administered every 12 h in the DOSE trial, and the daily dose was regarded as equal or high dose. This meant that a single IV furosemide dose in the DOSE trial was half of the daily equal or high dose. These facts indicated that the DOSE trial was inconclusive about the initial IV furosemide dose during the very acute phase of AHF.

### Strengths and limitations

The strength of our study is that we evaluated the dose of diuretics standardised by the amount of diuretic prescribed before admission. Previous studies that simply evaluated the diuretic dose prescribed during hospitalisation and the prognosis could be heavily confounded by the disease severity of the patients, i.e., those treated with a higher dose of diuretics were older, with a history of heart failure, and prescribed higher doses. This bias could not be fully adjusted, even if a multivariable model was applied. We found no such association in our study between the Above group and a high-risk profile.

There are also several limitations that should be acknowledged. This study was not predefined. It was a retrospective analysis of registry data; therefore, the results should be interpreted cautiously. Additionally, a significant number of patients were excluded for a late administration of the first IV furosemide because we focused on the very early phase of AHF treatment. Furthermore, the groups differed in some baseline characteristics. The worse outcome of the higher dose might reflect disease severity rather than the impact of the initial IV furosemide dose. The results consistently demonstrated an association between the initial IV furosemide dose and the outcome, even after adjusting for the Outcomes of a Prospective Trial of Intravenous Milrinone for Exacerbations of Chronic Heart Failure (OPTIME-CHF) score and catecholamine use to minimize the disease severity effect. However, we should emphasize that there could still be other unmeasured confounders owing to the retrospective nature of our study. Finally, since the dose of the diuretics in our study was relatively lower than previous studies, including the DOSE trial, whether such relatively low dose of the diuretics could have a significant effect on the mid-term mortality has been unclear. Further randomized studies are required to clarify the association between initial IV furosemide dose and prognosis.

## Conclusions

Treating patients with AHF with guideline-recommended initial IV furosemide dose was associated with shorter hospital stay than with lower doses and a higher 60-day survival rate than with higher doses. Our study results endorse the current guidelines concerning the first IV furosemide dose in terms of prognosis and diuretic efficiency.

## Methods

### Study design and patients

The present study utilised data from the REALITY-AHF, a prospective multicentre registry focused on the presentation and treatment during the very early phase of AHF hospitalisation. Details on the study design were published elsewhere^6^. Briefly, consecutive patients with AHF aged ≥ 20 years hospitalised through the emergency department (ED) in 20 hospitals in Japan were enrolled. The AHF diagnosis was determined by an attending physician at each site, using the Framingham criteria^12^. Patients with brain natriuretic peptide (BNP) < 100 ng/L or N-terminal pro b-type natriuretic peptide < 300 ng/L were excluded due to diagnostic uncertainty, following the guidelines^3^. Detailed inclusion and exclusion criteria and other study information are publicly available at the University Hospital Information Network (UMIN-CTR, unique identifier: UMIN000014105). Informed consent was obtained from all participants. The study protocol complied with the Declaration of Helsinki. It was first approved by the Kameda Medical Center, Clinical Research Committee (Kameda Medical Center, Research ethics committee), and subsequently approved by the ethical committee in each participating hospital before commencing patient enrolment (Tokyo Medical and Dental University, Research ethics committee; Nagoya University Graduate School of Medicine, Research ethics committee; St. Marianna University School of Medicine, Research ethics committee; Himeji Cardiovascular Center, Research ethics committee; Yokohama City University Medical Center, Research ethics committee; Fukushima Medical University, Research ethics committee; University of Tsukuba, Research ethics committee; the Sakakibara Heart Institute of Okayama, Research ethics committee; National Cerebral and Cardiovascular Center, Research ethics committee).

We analysed only patients treated with an IV bolus of furosemide within six hours of ED admission. Those treated with continuous furosemide infusion were excluded. We also excluded patients with hypotension (systolic blood pressure < 90 mmHg) at the time of ED admission. To validate the guideline-recommended initial IV furosemide dose, we divided the cohort into three dose groups (Below, Standard, and Above) according to whether the initial IV furosemide dose was lower, equal to, or higher than the guideline-recommended dose of 40 mg IV furosemide for patients with AHF not taking diuretics, or IV furosemide at the same dose as the oral loop diuretics for those already taking them^2,3^. Doses of other oral loop diuretics that were considered equivalent to 20 mg oral furosemide included 5 mg torasemide and 30 mg azosemide.

The primary endpoint was all-cause 60-day mortality. The evaluated secondary endpoints included DR and length of HS. Baseline data, including physical findings, echocardiography, and laboratory tests, were collected at the ED. A DR was defined as urine output (mL) obtained during the first six hours per 40 mg of IV furosemide (or equivalent).

### Statistical analysis

Data are presented as mean ± standard deviation or median and interquartile range (IQR) for continuous variables and as frequency (%) for categorical variables. One-way analysis of variance or the Kruskal–Wallis test was used to compare continuous variables. The χ^2^ or Fisher’s exact test was used to compare categorical variables. When necessary, variables were transformed for further analyses.

We performed univariate and multivariable linear regression analyses to evaluate the association between the first furosemide IV dose and DR and the length of HS. The multivariable model for DR was adjusted for age, whether the patient took oral loop diuretics before admission, white blood cell count, and serum levels of albumin, creatinine, and potassium, having already shown them to be independently associated with DR in this cohort^8^. The multivariable model for length of HS was adjusted according to the literature for age, sex, history of heart failure, the New York Heart Association (NYHA) class, systolic blood pressure, haemoglobin, serum levels of creatinine, sodium, and albumin, and log-transformed BNP. Moreover, atrial fibrillation was included in the multivariable analysis since it was reported to be associated with a blunted course of in-hospital decongestion in patients hospitalized with AHF^13^.

Event-free survival curves were constructed using the Kaplan–Meier survival method and compared using log-rank statistics. The OPTIME-CHF score was calculated for each patient as previously described^14^ to determine if the first furosemide IV dose was independently associated with mortality. The OPTIME-CHF risk score is based on age, the NYHA class, systolic blood pressure, and the levels of blood urea nitrogen and serum sodium. We used this score as an adjustment variable in the multivariable Cox model. Moreover, recent studies showed that BNP level at admission was associated with the prognosis^15^. Therefore, we also included the BNP level at admission as an adjustment variable.

All statistical analyses were performed using the R (The R Foundation for Statistical Computing, Vienna, Austria). In all analyses, a two-tailed *P*-value < 0.05 indicated statistical significance.

## Supplementary Information

Supplementary Information.

## Data Availability

The data underlying this article will be shared on reasonable request to the corresponding author.
