# Supplementary figures and images for: Clinical implication of initial intravenous diuretic dose for acute decompensated heart failure
Source: Sci Rep. 2022 Feb 8;12:2127. doi: 10.1038/s41598-022-06032-x (PMC8825846; doi:10.1038/s41598-022-06032-x)

Supplemental Figure S1


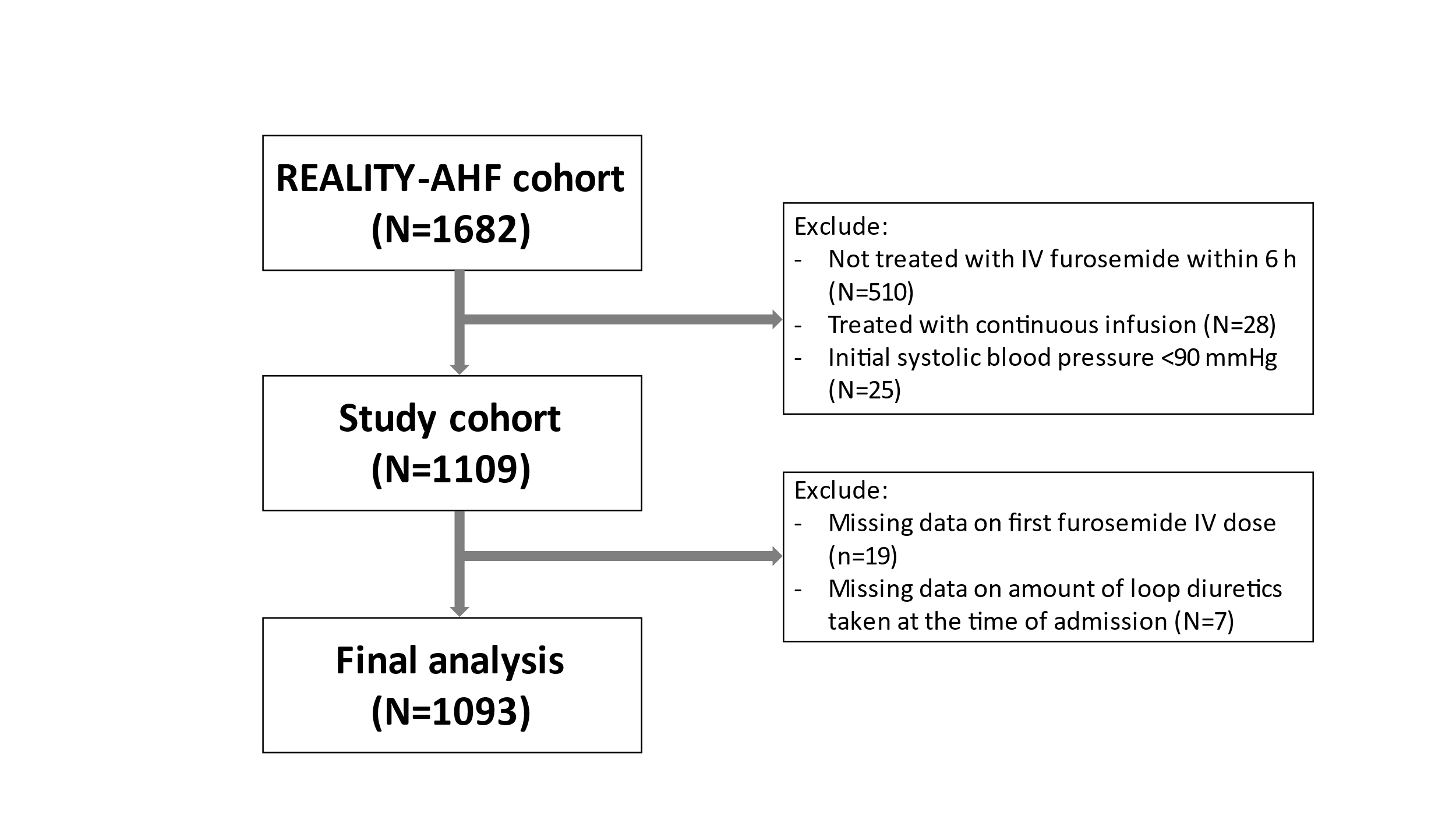


**Supplemental Figure S1**. A participant selection flow chart of the study

Supplement: Supplementary file 1 — Supplementary Information. [file 41598_2022_6032_MOESM1_ESM.docx]
